# Tuberculous uveitis: association between anti-tuberculous therapy and clinical response in a non-endemic country

**DOI:** 10.1186/s12348-017-0137-0

**Published:** 2017-10-04

**Authors:** Kristina L. Bajema, Kaivon Pakzad-Vaezi, Thomas Hawn, Kathryn L. Pepple

**Affiliations:** 1 Department of Medicine, Division of Allergy and Infectious Diseases, University of Washington, 1959 NE Pacific St., Box 356423, Seattle, WA 98195 USA; 2 Department of Ophthalmology, University of Washington, 325 9th Avenue, Box 359608, Seattle, WA 98104 USA

**Keywords:** Tuberculous uveitis, Ocular tuberculosis, *Mycobacterium tuberculosis*, Anti-tuberculous therapy

## Abstract

**Background:**

The study aims to report the association between successful uveitis control following anti-tuberculous therapy (ATT) for uveitis associated with a positive tuberculosis (TB) screening test in a low endemic setting. A retrospective chart review of cases between 2010 and 2017 at a tertiary uveitis referral center in the United States of America was conducted. Subjects with any form of uveitis, a positive TB interferon-gamma release assay or tuberculin skin test, and negative evaluation for other causes of uveitis were included. ATT was recommended in all cases and completed therapy was categorized as either adequate or inadequate for active TB infection. Location and severity of inflammation and the use of local versus systemic corticosteroid therapy was assessed at presentation and again after recommendation of ATT.

**Results:**

Thirty-one eyes of 20 individuals were identified. Uveitis activity improved in 22 eyes of 15 patients (13 treated adequately for active TB, 2 not adequately treated). Nine eyes of 5 patients did not have improved activity (1 adequately treated, 4 not adequately treated). All 9 individuals presenting with posterior or panuveitis who improved were adequately treated whereas the remaining 2 who did not improve were not (*P* 0.02). Among those with anterior or intermediate uveitis, no clear treatment patterns were observed between those who did and did not improve (*P* 0.50). Six individuals (30%) experienced significant ATT-related adverse effects.

**Conclusions:**

In a non-endemic setting, ATT for uveitis associated with a positive TB screening test may provide benefit in controlling ocular inflammation, particularly for those with posterior or panuveitis. The role for ATT in anterior or intermediate uveitis is less clear.

## Background

In 2014, 9.6 million people worldwide developed TB leading to 1.5 million deaths [1]. In the United States of America (USA), 9421 cases were reported during this period with 21% manifesting as extrapulmonary disease [2]. Ocular findings occur in 1–2% of patients with active TB.

Diagnosis of tuberculous uveitis (TBU) depends largely on history and characteristic exam findings [3]. Though ocular fluid can be sampled for acid-fast smear, culture, or *M. tuberculosis* polymerase chain reaction (MTB-PCR), this is often not performed due to low test sensitivity and morbidity related to an invasive procedure. Screening for past exposure to TB can also be done by an interferon-gamma release assay (IGRA) such as QuantiFERON-TB Gold (QFT-G). However, this cannot distinguish between latent and active infection, and while specificity is generally high in non-BCG-vaccinated individuals [4], less is known about test characteristics in the setting of TBU [5–7].

Successful outcomes in patients treated with ATT, when measured by improvement in inflammation or non-recurrence of inflammation, range from 47 to 89% [8]. This variability is due in part to heterogeneity in treatment practices as well as in clinical presentation. Though studies have suggested benefit of ATT for TBU, less is known about the role of therapy in different disease manifestations. To better understand the association between successful uveitis control in patients with presumed TBU and ATT in a low endemic setting, we report outcomes of 20 patients with a presumptive diagnosis seen at an academic referral center in the USA.

## Methods

We conducted a retrospective review of medical records of patients seen at the ophthalmology clinic at Harborview Medical Center at the University of Washington between 2010 and 2017. Individuals were included if ophthalmology assessment raised a concern for TBU. Specifically, criteria included individuals with clinical signs of uveitis; evidence of tuberculous infection by either tuberculin skin test (TST), QFT-G, or ocular sampling for MTB-PCR; and exclusion of non-tuberculous infectious and non-infectious etiologies by screening tests. Non-tuberculous etiologies included syphilis (syphilis IgG or *T. pallidum* particle agglutination assay (TPPA)), rheumatoid arthritis (rheumatoid factor (RF)), lupus (antinuclear antibodies (ANA)), sarcoidosis (angiotensin-converting enzyme (ACE) and chest radiography), and toxoplasmosis (toxoplasma IgM and IgG). All patients underwent independent clinical evaluation by an infectious disease specialist following referral from ophthalmology prior to initiation of ATT.

Data were abstracted from the De-identified Clinical Data Repository (DCDR) which contains information collected from the University of Washington clinical systems between 2010 and 2017. A Microsoft Amalga query was run on the DCDR database using the keyword search term “TB of the eye.” No additional cases of interest were captured by further searches which included tuberculous uveitis, tuberculous chorioretinitis, tuberculous conjunctivitis, tuberculous episcleritis, tuberculous interstitial keratitis, tuberculous iridocyclitis, and tuberculous keratitis. Several additional individuals receiving ongoing clinical care were also included in the study.

Abstraction of demographics (age, gender, country of origin), TB risk factors, prior active or latent TB infection (LTBI), medical co-morbidities, time from symptom onset to diagnosis, and site of involvement was performed using a standardized data tool. Site of inflammation was categorized by a fellowship-trained uveitis specialist as anterior uveitis, intermediate uveitis, posterior uveitis, or panuveitis according to Standardization of Uveitis Nomenclature (SUN) guidelines [9]. Unilateral or bilateral disease was also ascertained. Information collected on diagnostic evaluation included TST, QFT-G, ocular sampling for MTB-PCR, sputum sampling for acid-fast bacilli (AFB) smear and culture, chest radiograph or computed tomography (CT), and human immunodeficiency virus (HIV) test.

Information on recommended treatment was also documented and included anti-tuberculous drugs, systemic steroids, topical steroids, and any drug-related toxicities. Completed therapy was categorized by infectious disease-trained clinicians according to the following scheme: (1) Adequate active ATT (adequately treated for active TB): defined according to standard treatment for drug-susceptible pulmonary TB [10] and included individuals in whom a fluoroquinolone was substituted for ethambutol during the intensive phase. (2) Adequate alternative active ATT: this included patients who were either not started on a four-drug regimen or completed less than 2 months of intensive phase therapy but received at least 6 months of daily isoniazid- or rifampin-containing combined therapy. These treatment courses are sufficient for different types of paucibacillary disease such as culture negative pulmonary TB which is often treated with a shortened 4-month regimen per American Thoracic Society guidelines [10]. (3) Inadequate or no active ATT (inadequately treated for active TB). Inadequate active ATT did not meet any of the definitions above and included treatment for LTBI.

Finally, inflammation was reassessed after recommendation of ATT and improvement was determined using SUN criteria [9]. Improvement was defined for patients with anterior uveitis as decrease of anterior chamber (AC) cell by 2 grades or a final score of < 0.5+ with topical corticosteroid eye drops administered no more than two times daily (low-dose topical therapy). For patients with anterior and intermediate uveitis, a decrease in vitreous haze by two grades or a final score of < 0.5+ with low dose topical corticosteroid therapy or low-dose oral corticosteroid (< 10 mg daily) was considered improvement. For patients with posterior uveitis maintenance of quiescence of any active choroidal, retinal, or vascular lesions noted at presentation with low-dose oral corticosteroid (< 10 mg daily) therapy after an initial pulse and taper was considered improvement. Patients that required topical corticosteroid therapy > 2 drops daily, or corticosteroid sparing therapy with or without oral corticosteroids were designated not improved. Improvement was ascertained within a 1-month window of the date 6 months after cessation of ATT when possible. In the absence of long-term follow-up, the clinical exam at the last recorded follow-up was used. If patients declined ATT therapy, the study endpoint was determined at the office visit falling between 6 and 12 months after initiation of anti-inflammatory therapy. If improvement in inflammation was observed before initiation of ATT, treatment was reclassified as no ATT group for final analysis.

We used Fisher’s exact test to compare clinical improvement according to primary predictors of interest, adequate ATT and site of inflammation. To examine possible confounding effects, we used Fisher’s exact test to compare associations between clinical improvement and age (stratified as < 60 and ≥ 60 years), sex, birth in a high-burden country, duration of symptoms (stratified as ≤ 1 and > 1 year), and underlying immunosuppression. The study was reviewed and approved by the University of Washington Institutional Review Board.

## Results

Thirty-one eyes of 20 patients with TBU were identified. Full details regarding baseline demographic and clinical characteristics are presented in Table 1. Of note, 16 patients were born outside the USA with 7 being from high TB-burden countries [11]. Five patients had underlying immunosuppressing conditions: 1 with psoriasis receiving methotrexate; 2 with chronic hepatitis C; 1 with end-stage renal disease status post renal transplant on mycophenolate, prednisone, and tacrolimus; and 1 with diabetes.

**Table 1:** Demographic and clinical characteristics of patients diagnosed with tuberculous uveitis

Baseline characteristics	
Median age (IQR), years	38 (31–58)
Men, *n*/*N* (%)	12/20 (60%)
Foreign born, *n*/*N* (%)	16/18 (89%)
Born in high TB-burden country	7/18 (39%)
Prior clinical history of active TB, *n*/*N* (%)	3/20 (15%)
Prior clinical history of LTBI, *n*/*N* (%)	4/20 (20%)
Median time from onset of symptoms to ATT (IQR), months	24 (3–96)
Positive screening test for LTBI	20/20 (100%)
QFT-G	18
TST	2
Abnormal chest imaging, *n*/*N* (%)^a^	2/19 (11%)
HIV test negative, *n*/*N* tested (%)	14/14 (100%)
Active concurrent TB, *n*/*N* (%)	0/20 (0%)

^a^Active pulmonary TB excluded in both cases with negative sputum tests for AFB smear, mycobacterial culture, and MTB-PCR

Eleven patients presented with bilateral disease while 9 had unilateral findings. Six presented with anterior uveitis, 3 had anterior and intermediate uveitis, 3 presented with posterior uveitis, and 8 had panuveitis. Alternative etiologies for uveitis were excluded as described in “Methods”.

ATT was initiated in 17 of 20 individuals (Table 2). Fourteen received four drugs for at least part of their therapy. Median duration among those completing therapy was 6 months (IQR range 6–8 months). All patients were treated with topical, local, or systemic corticosteroids to achieve inflammation quiescence per consensus treatment guideline for patients with uveitis [9]. Twelve individuals required treatment with systemic steroids for initial inflammation control. After inflammation control was achieved, corticosteroid tapering was initiated at the treating physician’s discretion but typically proceed by 10 mg per day per week for daily systemic doses > 20 mg/day and by 2.5–5 mg/day/week for doses between 5 and 20 mg/day. Topical therapy was tapered by one drop/day/week once AC cell was improved to 1+ cell or less, and as long as quiescence was maintained.

**Table 2:** Clinical presentation, treatment details, and clinical outcomes of 20 study patients

Case	Sex	Uveitis presentation	Unilateral or bilateral	Treatment regimen started	Duration therapy completed (months)	Additional systemic steroids	Uveitis improved
1	M	Panuveitis^a,b^	Bilateral	RH^d^	6	No	Yes
2	M	Posterior uveitis	Unilateral	RHZM	8	No	Yes
3	F	Panuveitis	Unilateral	RHZ	6	No	Yes
4	M	Anterior uveitis	Unilateral	RHZE	6	No	Yes^e^
5	F	Panuveitis^a^	Unilateral	RHZE	6	Yes	Yes
6	M	Anterior uveitis	Unilateral	–	–	No	Yes
7	F	Anterior uveitis	Bilateral	RHZE	18	Yes	No
8	M	Posterior uveitis^b,c^	Bilateral	–	–	Yes	No
9	M	Panuveitis	Unilateral	RHZE	9	Yes	Yes
10	M	Anterior uveitis^c^	Bilateral	RHZE	6	Yes	Yes
11	F	Panuveitis^a,b^	Bilateral	RHZE	6	Yes	Yes
12	M	Posterior uveitis^b^	Unilateral	RHZM	5	Yes	Yes
13	F	Anterior, intermediate uveitis^a^	Bilateral	RHZM	9	No	Yes
14	F	Anterior, intermediate uveitis^a^	Bilateral	RHZM	6	No	Yes
15	F	Panuveitis^a,c^	Bilateral	RHZE	Sporadic	Yes	No
16	M	Anterior, intermediate uveitis^b^	Bilateral	–	–	Yes	No
17	M	Anterior uveitis^a^	Unilateral	RHZE	7	No	Yes
18	F	Anterior uveitis	Unilateral	RPTH	3	Yes	No
19	M	Panuveitis uveitis	Bilateral	RHZE	6	Yes	Yes
20	M	Panuveitis	Bilateral	RHZL	7	Yes	Yes

^a^Cases born in a high TB-burden country: 1, 5, 11, 13, 14, 15, 17

^b^Cases with underlying immunosuppression: 1, 8, 11, 12, 16

^c^Cases with reported prior active TB: 8, 10, 15

^d^ATT abbreviations: R—rifampin, RPT—rifapentine, H—isoniazid, Z—pyrazinamide, E—ethambutol, M—moxifloxacin

^e^Inflammation improved before the start of ATT

**Fig. 1:**
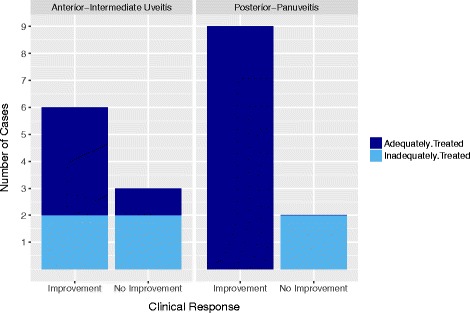
Clinical improvement stratified by initial uveitis presentation and adequate or inadequate ATT for active TB. Numbers presented as individuals

At the study endpoint, 22 eyes of 15 patients were improved—20 eyes of 13 patients who were adequately treated for active TB and 2 eyes of 2 patients who were not. In 1 of the untreated cases, inflammation improved before ATT was initiated. Nine eyes of 5 patients were not improved; of these, only 2 eyes in 1 patient were adequately treated while 7 eyes in the remaining 4 patients were not (1 patient treated for LTBI, 1 inadequately treated, and 2 not treated; *P* 0.01 comparing proportion improved in adequate versus inadequate ATT groups). All 9 individuals presenting with posterior or panuveitis who improved were adequately treated whereas the remaining 2 who did not improve were not (1 inadequately treated, 1 untreated; *P* 0.02) [Fig. 1]. Among 9 patients with anterior or intermediate uveitis, no clear treatment patterns were observed between those who did and did not improve though 1 improved without any therapy (*P* 0.50). There was no association between clinical improvement and age, sex, birth in a high-burden country, duration of symptoms, or underlying immunosuppression.

Adverse drug effects from ATT were common. While 4 developed only mild symptoms, 6 (30%) had severe reactions or toxicities requiring a temporary or permanent cessation of the offending agent. These included 2 individuals with drug-induced hepatitis, 1 with severe allergic reaction, and 3 with nausea and vomiting.

## Discussion

In a non-endemic setting, we found that patients with posterior or panuveitis who received adequate ATT were able to achieve ocular inflammation control more often than patients that did not have adequate treatment. In contrast, no such association was seen in patients with anterior and intermediate uveitis.

Ocular TB is thought to represent either active mycobacterial infection or a delayed-hypersensitivity reaction to bacilli present at a different site in the body. Support for the former hypothesis is based on studies of patients with ocular inflammation in which *M. tuberculosis* was identified in ocular specimens through histopathology or nucleic acid amplification [12–14]. While ATT is indicated for active infection, it is not expected to be of therapeutic benefit in treating an immune-mediated reaction. The hypothesis of active infection is supported by studies that demonstrate better clinical response in the setting of longer ATT duration. Compared to no treatment, ATT for more than 9 months was associated with a significant reduction in recurrence of inflammation [15], an effect than was not significant in shorter treatment durations. In another study, the risk of inflammation recurrence was significantly reduced among those receiving more than 9 months of ATT compared with shorter course therapy [16]. Other studies of treatment response are more equivocal with regard to the hypothesis of active infection. In a meta-analysis by Kee et al., the pooled relative risk of ATT for improvement in inflammation related to TBU was only 1.17 (95% CI 0.74–1.85) and 1.42 (95% CI 1.24–1.63) for non-recurrence of inflammation. Thus the role for ATT in TBU remains unclear. Our study suggests that multi-drug therapy for active TB provides higher cure rates than interrupted regimens or no treatment. Taken together, our data support a model of active infection as opposed to a pure hypersensitivity response to *M. tuberculosis* antigens in the absence of live bacilli.

In our cohort, individuals with posterior or panuveitis were more responsive to ATT than those with anterior or intermediate uveitis. Few studies have examined treatment outcomes in the context of differing sites of ocular inflammation. Ang et al. showed a trend toward recurrence of inflammation following ATT comparing intermediate to anterior uveitis as well as non-significant reduction in recurrence of inflammation comparing posterior to anterior uveitis [15]. In contrast, Patel et al. described an increased risk of relapse comparing posterior to other forms of uveitis including panuveitis [17]. However, the authors noted that relapse occurred less often among those receiving a full ATT course and that relapsed cases responded to extended therapy. In a large retrospective study of 360 patients with uveitis and a positive TB screening test, Bansal et al. also reported an overall significant reduction in recurrence of inflammation when ATT was added to corticosteroid therapy compared to corticosteroids alone [18]. Though anatomic location of uveitis was not found to be a significant predictor of recurrence risk, their data is consistent with the trend identified here; patients with anterior or intermediate uveitis are more likely to have continued disease activity despite combined treatment with ATT and corticosteroids. Although different treatment approaches are not routinely employed based on the location of ocular inflammation, future studies should address this possibility. In particular, anterior and intermediate uveitis may require different treatment than other forms of uveitis.

Adverse drugs effects related to ATT were common; one third of treated patients experienced severe toxicities that required either temporary or permanent drug discontinuation. This is much higher than what has been reported in previous studies [8] and may reflect differences in age, co-morbidities, and toxicity monitoring.

Our study is limited by small numbers, heterogeneity in ATT and corticosteroid regimens, and need for long-term follow-up to evaluate recurrence of inflammation. Though ATT was classified dichotomously as adequate or inadequate for active TB, LTBI or partial treatment courses can be expected to have some benefit in paucibacillary infection. As only two patients were treated in this manner, the impact on interpretation of treatment-related outcomes was minimal. Incidental lack of ATT in some cases did afford the opportunity to observe treatment-independent responses which has not been extensively reported in the literature. Altogether, these findings suggest a role for ATT among individuals with posterior uveitis and panuveitis. Lack of clear benefit in anterior and intermediate uveitis combined with high risk of adverse events may warrant a period of observation before considering ATT. Our study also lacks a control group of uveitis patients without a positive TB screening test; therefore, conclusions about the benefit of ATT on uveitis control and the mechanism underlying any benefit are limited. Ultimately, a randomized trial is needed to fully address this question.

## Conclusions

In agreement with prior studies in paitents with uveitis in TB endemic retions, in a TB non-endemic setting, ATT for uveitis associated with a positive TB screening test may provide benefit in controlling ocular inflammation, particularly for those with posterior or panuveitis. The role for ATT in anterior or intermediate uveitis is less clear.
